# The Diagnostic Yield of Laboratory Tests in Chronic Constipation in Adults

**DOI:** 10.7759/cureus.66116

**Published:** 2024-08-04

**Authors:** Ali A Ramadhan, Aveen Mustafa, Ramadhan Issa, Hassan Bapeer

**Affiliations:** 1 Department of Gastroenterology and Hepatology, University of Duhok, Duhok, IRQ; 2 College of Medicine, University of Duhok, Duhok, IRQ

**Keywords:** adults, duhok, chronic constipation, laboratory tests, diagnostic yield

## Abstract

Background: Chronic constipation is a common gastrointestinal complaint characterized by infrequent or difficult bowel movements, significantly affecting patients' quality of life. Laboratory markers offer potential diagnostic value in identifying physiological changes associated with chronic constipation, yet their effectiveness remains underexplored.

Objectives: The objective of this study was to evaluate the diagnostic value of various laboratory tests in identifying the underlying causes of chronic constipation among adults.

Patients and methods: A cross-sectional study was conducted at Kurdistan Private Hospital and Jeen Clinics in Duhok, Kurdistan, Iraq, from December 2022 to May 2024. A total of 132 patients meeting the Rome IV criteria for chronic constipation were included. Data collection involved demographic information, lifestyle factors, and laboratory tests, including complete blood count (CBC), thyroid stimulating hormone (TSH), serum calcium, serum potassium, serum glucose, serum creatinine, parathyroid hormone (PTH), and vitamin D levels.

Results: The study population consisted of 56 males (42.4%) and 76 females (57.6%) with a mean age of 46.5 years (SD=17 years) and a range of 18-81 years. Regular exercise was performed by only 56 (42.4%) patients, 85 (64.4%) patients were drinking less than 2 liters of water per day, and 108 (81.8%) were overweight or obese. Of the study population, hypothyroidism was detected in 27 (20.4%), hyperparathyroidism in 27 (20.4%), anemia in 58 (44%), leukocytosis in 24 (18.2%), renal impairment in 48 (36.4%), hypokalemia in four (3%), hyperkalemia in 12 (9.1%), hypocalcemia in 10 (7.6%), hypercalcemia in 12 (9.1%), impaired fasting glucose in 46 (34.8%), hyperglycemia in 21 (15.9%), and vitamin D deficiency in 80 (60.6%). Of the study population, 40 (30%) patients had normal laboratory investigations panel.

Conclusion: In chronic constipation, laboratory tests have high diagnostic yield in adults and are essential for ruling out secondary causes of chronic constipation. Unhealthy lifestyles are prevalent in patients with chronic constipation.

## Introduction

Chronic constipation is a widespread gastrointestinal condition presented by infrequent bowel movements (typically fewer than three per week) and difficulty in passing stools. Patients usually complain of symptoms, such as straining, hard stools, abdominal discomfort, and bloating, with a sensation of incomplete evacuation [1].

The global prevalence of chronic constipation is estimated to be between 15% and 20%, leading to numerous outpatient visits and substantial healthcare costs [2]. Although it is common, chronic constipation often remains under-recognized until patients present with complications like anorectal disorders [3]. 

Generally, the etiology of chronic constipation can be subdivided into primary or secondary. The exact mechanism of primary constipation is not well understood but is thought to be due to disturbances in the neuromuscular function of the colon or the anorectal neurosensory coordination. Primary constipation is further subdivided into subtypes including normal transit constipation, slow transit constipation, and disorders of defecation (outlet dysfunction). The distinction between these is based on radio-opaque marker study and anorectal manometry [4,5]. Secondary chronic constipation can result from many causes like metabolic disorders, medications, or organic causes. Diabetes, hypothyroidism, hyperparathyroidism, and electrolyte disturbances are among the important metabolic causes. The common medications contributing to constipation are antihypertensives like calcium channel blockers, analgesics like opioids, and anticholinergics like antidepressants. The most important organic disease to be excluded is colorectal cancer, although other structural abnormalities can cause constipation [6].

The management of chronic constipation can be challenging but using the systematic diagnostic evaluation process that aims at identifying the exact cause of constipation may be rewarding. This process should start with detailed history taking followed by a proper physical examination. The role of laboratory investigations comes next but the detailed list of laboratory investigations to be requested in patients with chronic constipation is still a matter of debate until now. Tailoring laboratory testing to the results of the clinical assessment may miss several conditions, especially since many metabolic or structural diseases can present with non-specific findings. Complete blood count is advisable in all patients but the use of tests for thyroid stimulating hormone, parathyroid hormone, or serum levels of glucose, creatinine, calcium, and potassium is pending validation, although many clinicians incorporate these tests into the initial evaluation of patients with chronic constipation [7]. This indeterminate level of evidence in the current recommendations was a great impulse for us to conduct this study aiming at evaluating the diagnostic yield of laboratory tests in chronic constipation among adults and identifying the most effective laboratory set as we think that this would contribute to more effective diagnostic evaluation, which will ultimately translate into better management outcomes and patient welfare [7,8].

## Materials and methods

This was a cross-sectional study conducted at the Kurdistan Private Hospital and Jeen Clinics in Duhok, Iraq, over a period of 18 months from December 2022 to May 2024. The study protocol was approved by the Ethics Committee of the College of Medicine, University of Duhok, Ministry of Higher Education & Scientific Research, Kurdistan Regional Government, Iraq (approval number: 1243Q). The study aims and the protocol was clarified to the patients and informed consent was obtained.

The study included 132 patients who met the Rome IV criteria for chronic constipation (Table 1) and were aged 18 years and older. Exclusion criteria included patients with evidence of acute illness, patients on chronic medications known to affect bowel habits (e.g., laxatives, opioids), and pregnant women.

**Table 1:** Diagnostic criteria for chronic constipation IBS: irritable bowel syndrome Reference: Rome Foundation [9]

Criterion	Description
Symptom Duration	Symptoms present for the last three months with onset at least six months prior to diagnosis
Frequency	Fewer than three bowel movements per week
Stool Consistency	Hard or lumpy stools in more than ¼ (25%) of defecations
Straining	Straining during more than ¼ (25%) of defecations
Incomplete Evacuation	Sensation of incomplete evacuation in more than ¼ (25%) of defecations
Anorectal Obstruction/Blockage	Sensation of anorectal obstruction/blockage in more than ¼ (25%) of defecations
Manual Maneuvers	Manual maneuvers to facilitate more than ¼ (25%) of defecations (e.g., digital evacuation, support of the pelvic floor)
Use of Laxatives	Loose stools are rarely present without the use of laxatives
Insufficient Criteria for IBS	Symptoms do not fully meet IBS criteria but indicate a functional gastrointestinal disorder

The data collected from the eligible patients included demographic information (age, sex), duration of constipation, and lifestyle habits (exercise, water intake, smoking) and then anthropometric measurements were taken to calculate the body mass index. After that, a 5 ml sample of venous blood was aspirated without tourniquet use under an aseptic technique to measure the CBC, TSH, PTH, calcium levels, potassium levels, fasting serum glucose levels, creatinine, and vitamin D levels. The CBC was measured using an automated hematology analyzer (Coulter counter; Sysmex Corporation, Kobe, Hyogo, Japan) and the biochemical tests were measured using cobas c 111 (F. Hoffmann-La Roche AG, Basel, Switzerland). 

Obesity was classified depending on body mass index (BMI) using the World Health Organization (WHO) classification [10]. The normal hemoglobin ranges are 14-18 g/dL for males and 12-16 g/dL for females. The normal WBC count was taken as 4000-11000 cells/µL [11]. The manufacturer identified the normal ranges for TSH (0.4-4 mU/L), PTH (15-65 pg/mL), creatinine (0.6-1.2 mg/dl), calcium (8.5-10.2 mg/dL), potassium (3.6-5.2 mmol/L) and fasting serum glucose (70-100 mg/dl). The serum levels of vitamin D were considered deficient (< 20 ng/mL), insufficient (20-29 ng/mL), or normal (30-100 ng/mL) [12].

Data analysis was done with the aid of IBM SPSS Statistics for Windows, Version 24.0 (Released 2016; IBM Corp., Armonk, NewYork, United States). For continuous variables, the mean and standard deviation (SD) were calculated while categorical variables were expressed as frequencies and percentages. 

## Results

Of the 132 patients included in the study, 76 (57.6%) were females with the female-to-male ratio being 1.3:1. The age distribution indicates that chronic constipation affects a wide range of ages but 98 (74.2%) participants were younger than 60 years. The duration of constipation was on average 1.5 years with 107 (81%) of the study population having constipation for up to five years before doing medical consultation. Physical exercise was not done by 76 (57%) patients and 39 (29.5%) exercised for less than 30 minutes. Of the study population, 85 (64.4%) had an average water intake per day of less than 2 liters and the same number were non-smokers. According to WHO classification, 59 (44.7%) of patients were overweight and 40 (30.3%) were obese class-I. With regard to Rome IV criteria, 131 (99.2%) were complaining of less than three bowel motions per week, 47 (35.6%) had a sensation of incomplete evacuation in >25% of defecations, 43 (32.6%) had hard or lumpy stools in > 25% of defecations, 41 (31.1%) had sensation of anorectal obstruction in >25% of defecations, 25 (19%) had straining on >25% of defecations, and only 22 (16.7%) were using manual maneuvers in >25% of defecations. This is shown in Table 2.

**Table 2:** Demographic and clinical characteristics of study population (N=132) Values given in number and percentage and mean ± SD (where marked)

Parameters		Number	Percentage
Sex	Female	76	57.6%
	Male	56	42.4%
Age in years	Mean ± SD	46.5 ± 17	
	Age range	18-81	
Duration of constipation in years	Mean ± SD	1.5 ± 0.4	
	<1 year	42	31.8%
	1-5	65	49.2%
	5-10	19	14.4%
	>10	6	4.5%
Physical exercise	No physical exercise	76	57.6%
	< 30 minutes /day	39	29.5%
	30-60 minutes/day	14	10.6%
	> 60 minutes /day	3	2.3%
Average water intake	< 0.5 L/day	14	10.6%
	0.5-1 L/day	29	22 %
	1-2 L/day	42	31.8%
	> 2 L/day	47	35.6%
Smoking status	Current smokers	25	18.9%
	Ex-smokers	22	16.7%
	Non-smokers	85	64.4%
Body mass index (Kg/m^2^)	Mean ± SD	28.6 ± 4.6	
	Underweight	3	2.3%
	Normal weight	21	15.9%
	Overweight	59	44.7%
	Obesity Class 1 (Mild)	40	30.3%
	Obesity Class 2 (Moderate)	8	6%
	Obesity Class 3 (Severe)	1	0.8%
Patients meeting Rome IV clinical criteria	< 3 spontaneous movements /week	131	99.2%
	Straining in > 25% of defecations	25	19%
	Hard stools in > 25% of defecations	43	32.6%
	Sensation of incomplete evacuation in >25% of defecations	47	35.6%
	Sensation of anorectal obstruction in >25% of defecations	41	31.1%
	Manual maneuvers in >25% of defecations	22	16.7%

Of the study participants, hypothyroidism was detected in 27 (20.4%), hyperthyroidism in four (3%), hyperparathyroidism in 27 (20.4%), hypoparathyroidism in three (2.3%), anemia in 58 (44%), polycythemia in 1 (0.8%), leukocytosis in 24 (18%), leukopenia in 4% (3%), renal impairment in 48 (36%), hypocalcemia in 10 (7.6%), hypercalcemia in 12 (9%), hypokalemia in four (3%), hyperkalemia in 12 (9%), impaired fasting glucose in 46 (34.8), hyperglycemia in 21 (15.9%), and vitamin D deficiency in 80 (60.6%). Forty (30%) patients had normal laboratory investigations panel. This is shown in Table 3.

**Table 3:** Laboratory findings in the study population (N=132) TSH: thyroid-stimulating hormone; PTH: parathyroid hormone

Investigations		Number	Percentage
TSH	Hyperthyroidism (<0.4 mU/L)	4	3%
	Euthyroid (0.4-4 mU/L)	101	76.5%
	Subclinical hypothyroidism (4-10 mU/L)	23	17.4%
	Overt hypothyroidism (>10.0 mU/L)	4	3%
PTH	Hypoparathyroidism (<15 pg/mL)	3	2.3%
	Normal (15 to 65 pg/mL)	102	77.3%
	Hyperparathyroidism (>65 pg/mL)	27	20.4%
Hemoglobin	Anemia	58	43.9%
	Normal hemoglobin	73	55.3%
	Polycythemia	1	0.8%
While blood cells	Leucopenia (<4,000 cells/µL)	4	3%
	Normal (4,000 to 11,000 cells/µL)	104	78.8%
	Leukocytosis (>11,000 cells/µL)	24	18.2%
Creatinine	Below normal (<0.6 mg/dL)	3	2.3%
	Normal (0.6 - 1.2 mg/dL)	81	61.4%
	Above normal (>1.2 mg/dL)	48	36.4%
Calcium	Hypocalcemia (<8.5 mg/dL)	10	7.6%
	Normal (8.5 to 10.2 mg/dL)	110	83.3%
	Hypercalcemia (>10.2 mg/dL)	12	9.1%
Potassium	Hypokalemia (<3.6 mmol/L)	4	3%
	Normal (3.6-5.2 mmol/L)	116	87.9%
	Hyperkalemia (>5.2 mmol/L)	12	9.1%
Glucose	Below normal (<70 mg/dL)	3	2.3
	Normal (70-100 mg/dL)	62	47%
	Impaired fasting glucose (100-125 mg/dl)	46	34.8%
	Hyperglycemia (>125 mg/dL)	21	15.9%
Vitamin D	Mean ± SD	16.3±8.5	
	Deficiency (<20 ng/mL)	80	60.6%
	Insufficiency (20-29 ng/mL)	23	17.4%
	Normal (20-100 ng/mL)	29	22%

## Discussion

Chronic constipation is a frequent digestive disorder with a significant impact on the patient’s quality of life. The condition is multi-factorial and could be primary or secondary. The differentiation between these depends mainly on the investigations used to identify the secondary causes. Colonoscopy and imaging have been used to identify structural diseases but the use of laboratory investigations to identify metabolic dysfunctions associated with constipation has been a matter of debate and their diagnostic value has been questioned as the American Gastrointestinal Association's medical position statement states that the diagnostic utility of laboratory investigations have not been rigorously evaluated but is probably low [13,14]. This urged us to revise the diagnostic yield of laboratory investigations in patients presenting with chronic constipation as this might enhance the ability to identify secondary causes and adjust treatment plans for patients suffering from this disorder.

The majority of this study population were females which is comparable to that revealed by other studies [13]. This could probably be due to increased hormonal factors during the estrogenic phase of the menstrual cycle [15]. Almost three out of four patients in this study were younger than 60 years, which is contrary to that shown in other studies [13]. This can be explained by the fact that younger individuals may be more likely to seek medical help to identify the underlying cause of their constipation while elderly patients are more likely to have multiple diseases and drugs that could at least explain to them the cause of their constipation without consultation.

The average duration of constipation before patients sought medical consultation was 1.5 years and this might reflect the non-urgency of constipation as a clinical condition and the fact that many patients use over-the-counter medications and seek medical help only when the condition becomes persistent [13]. Studies have shown that lack of physical exercise and inadequate water intake have been associated with constipation [16,17] and these findings have been replicated in this study also. The link between smoking and constipation is inverse so stopping smoking might initiate or increase constipation as it is thought that nicotine has a laxative effect [18]. This also has been noted in this study as most of the study population were non-smokers. Constipation has been associated with obesity in many studies [19], and this was also observed by this study. Whether this reflects a primary pathophysiological dysfunction or is secondary to other factors like physical inactivity or associated diabetes remains largely unknown and can be a subject for further study [19].

Even after an extensive web search, we could not find any study that directly addressed the subject of laboratory testing in chronic constipation in adults. A study done in children concluded low diagnostic yield and high cost for the use of screening tests in constipated children [20] but to replicate the results of such a study in the adult population without specific studies in adults seems non-logical as the two populations are different from many perspectives.

This study could identify a laboratory abnormality in two out of the three patients being evaluated for chronic constipation by laboratory testing and this constitutes a high diagnostic yield. The actual contribution of abnormal laboratory testing, its cost effectiveness, and its impact on constipation treatment remains to be identified and could be the subject of future studies. But even if the abnormal tests have no direct correlation with the cause or treatment of constipation, it remains an opportunity to detect conditions like vitamin D deficiency, subclinical hypothyroidism, or impaired fasting glucose which could impact the patients’ quality of life in the future. 

The most frequent abnormality detected was vitamin D deficiency and the association between constipation and vitamin D deficiency has been confirmed in other studies including one conducted by Panarese et al. [21]. Vitamin D deficiency accounted for more than half of the cases of hyperparathyroidism (15 out of 27 cases). In the literature, hypothyroidism, hyperparathyroidism, hypercalcemia, hypokalemia, kidney disease, and diabetes are stated as causes of secondary constipation [14], and in this study, we were able to detect laboratory abnormalities indicative of these endocrine and metabolic diseases in more than one third (53 patients, 40%) of the study population. Although anemia is not a cause of constipation, it may be a feature for the other causes of constipation like kidney disease, and is an alarm feature that should be followed by more intensive investigations including colonoscopy [13]. The actual contribution of other findings like leukopenia and leukocytosis to constipation will need further study.

This study has limitations including the small sample size and its cross-sectional nature that included a specific group of patients. Nevertheless, the results of this study put an exclamation mark on the recommendations stated by agencies like the American Gastrointestinal Association which underestimates the role of laboratory investigations in chronic constipation without strong evidence and calls for more studies in this regard including large-scale population-based studies that might include more markers that could increase our depth of understanding the pathophysiology of constipation and lead to better management plans with resultant reduction in the suffering of patients with this common condition. 

## Conclusions

In chronic constipation, laboratory tests have a high diagnostic yield in adults and are essential for ruling out secondary causes of chronic constipation. More judicious use of these tests in the evaluation process of patients with chronic constipation may result in earlier diagnosis of diseases with subtle clinical features like hypothyroidism. Unhealthy lifestyles are prevalent in patients with chronic constipation and it is important to consider this in the initial evaluation of the patient and also in the management plan in order to achieve optimal outcomes and improve the patient's quality of life. 
